# Treatment of Alzheimer's Disease with Deep Cervical Lymph‐Venous Anastomosis

**DOI:** 10.1002/alz70858_100207

**Published:** 2025-12-25

**Authors:** Mian Guo, Nannan Zhao, Qingsong Yang, Fan Fan

**Affiliations:** ^1^ Harbin Medical University, Harbin, Heilongjiang, China; ^2^ Augusta University, Augusta, GA, USA

## Abstract

**Background:**

Alzheimer's Disease (AD) is a progressive neurodegenerative disorder with no cure. AD is characterized by the pathological accumulation of Aβ plaques and hyperphosphorylated tau proteins. Recent studies have found that the lymphatic system plays a crucial role in the clearance of Aβ and tau. The present study seeks to explore whether deep cervical lymph‐venous anastomosis (LVA), a surgery that was initially for the treatment of cervical lymphatic obstruction, has beneficial effects in AD patients.

**Methods:**

This study included 65 AD patients who met the 2024 diagnostic criteria and were in stages 4 to 6 of the disease, determined by the Montreal Cognitive Assessment (MoCA), Mini‐Mental State Examination (MMSE), and Activities of Daily Living (ADL). After anesthesia, indocyanine green (10 mM, 0.1 mL) was injected submucosally into the bilateral middle nasal turbinate to visualize deep cervical lymphatic vessels. The internal jugular vein was exposed, and fluorescently highlighted adjacent lymph nodes were carefully selected. Two‐thirds of the lymph nodes were excised, while the remaining portion was anastomosed end‐to‐side with the internal jugular vein. A drainage tube was placed and removed on postoperative day 1. The procedure was performed bilaterally. MoCA, MMSE, and ADL were assessed on days 7 and at 3, 6, and 12 months post‐operation and compared to preoperative baselines.

**Results:**

In 65 deep cervical LVA AD cases, nearly 90% showed overall improvements in cognition, daily living abilities, and quality of life. By postoperative day 7, most patients exhibited cognitive improvement accompanied by excitement, insomnia, or irritability. By postoperative 3 months, cognitive function and daily living abilities had all significantly improved. Ongoing studies are continuing to track longer‐term outcomes.

**Conclusion:**

The present study provided strong evidence demonstrating that deep cervical LVA improves cognitive function, mental state, and daily activities in AD patients. Our results suggest that this surgical procedure offers a promising therapeutic approach for AD treatment and paves the way for further investigation into its long‐term consequences and underlying mechanisms.

